# Experimental Infection of Calves with *Escherichia coli* O104:H4 outbreak strain

**DOI:** 10.1038/srep32812

**Published:** 2016-09-07

**Authors:** K. Hamm, S. A. Barth, S. Stalb, L. Geue, E. Liebler-Tenorio, J. P. Teifke, E. Lange, K. Tauscher, G. Kotterba, M. Bielaszewska, H. Karch, C. Menge

**Affiliations:** 1 Friedrich-Loeffler-Institut, Institute of Molecular Pathogenesis, Naumburger Str. 96a, 07743 Jena, Germany; 2 Friedrich-Loeffler-Institut, Department of Experimental Animal Facilities and Biorisk Management, Südufer 10, 17493 Greifswald – Insel Riems, Germany; 3 Friedrich-Loeffler-Institut, Institute of Infectology, Südufer 10, 17493 Greifswald – Insel Riems, Germany; 4 Institute of Hygiene, University of Münster, Robert-Koch-Straße 41, 48149 Münster, Germany

## Abstract

In 2011, a severe outbreak of hemolytic-uremic syndrome was caused by an unusual, highly virulent enterohemorrhagic *E. coli* (EHEC) O104:H4 strain, which possessed EHEC virulence traits in the genetic background of human-adapted enteroaggregative *E. coli*. To determine magnitude of fecal shedding and site of colonization of EHEC O104:H4 in a livestock host, 30 (ten/strain) weaned calves were inoculated with 10^10^ CFU of EHEC O104:H4, EHEC O157:H7 (positive control) or *E. coli* strain 123 (negative control) and necropsied (4 or 28 d.p.i.). *E. coli* O157:H7 was recovered until 28 d.p.i. and O104:H4 until 24 d.p.i. At 4 d.p.i., EHEC O104:H4 was isolated from intestinal content and detected associated with the intestinal mucosa. These results are the first evidence that cattle, the most important EHEC reservoir, can also carry unusual EHEC strains at least transiently, questioning our current understanding of the molecular basis of host adaptation of this important *E. coli* pathovar.

In early May 2011, an unprecedented large outbreak of hemolytic-uremic syndrome (HUS) and hemorrhagic colitis (HC) started in Germany and subsequently spread throughout Europe and North America. Nearly 4,000 individuals were affected, including almost 900 cases of HUS and 54 deaths^1^. The outbreak was linked to the consumption of contaminated sprouted fenugreek seeds^2^. An unusual enterohemorrhagic *Escherichia coli* (EHEC) strain of serotype O104:H4 was identified as the causative agent^1^. The strain possesses a combination of virulence factors from both Shiga toxin (Stx)-producing *E. coli* (STEC) and enteroaggregative *E. coli* (EAEC) strains^3^,^4^ and represents a novel hybrid pathotype of enterohemorrhagic *E. coli* (EHEC).

EHEC, the human pathogenic subset of STEC, has emerged as an important cause of human disease in developed countries^5^. The pathogens are transmitted to humans through consumption of raw or undercooked food or water contaminated by ruminant feces^6^ or via direct contact with infected persons or animals^7^. Clinical symptoms of EHEC infections range from mild diarrhea to HC^8^ and may be complicated by the potentially fatal HUS^9^. Characterization of the epidemic EHEC O104:H4 strain showed that it carries *stx*_2_ encoding for the eponymous virulence factor of STEC but, in contrast to typical EHEC, lacks the locus of enterocyte effacement (LEE). EHEC O104:H4 instead harbors genetic markers of EAEC (*aatA*, *aggA*, *aggR*, *aap*) located on the EAEC pAA virulence plasmid and expresses the corresponding phenotype of aggregative adherence to intestinal epithelial cells^3^,^10^. Typical EAEC are commonly associated with acute and persistent (>14 days) diarrhea in children^11^, immunosuppressed persons^12^, and travelers to developing countries^13^, and with some cases of diarrhea in developed countries^14^.

The principal reservoir of STEC strains associated with human disease are ruminants. Several bacterial factors (e.g., intimin^15^, other products of the LEE^16^, and Efa-1^17^) promote bacterial colonization of the intestinal mucosa resulting in attaching and effacing lesions of *E. coli* O157:H7 on epithelial cells. Stxs primarily delay the host’s cellular immune response, thereby generating an opportunity for STEC bacterial colonization^18^. Rectum and ileocecal valve (ICV) are sites most likely to contain STEC O157:H7 bacteria^19^,^20^,^21^. STEC can be regularly detected in cattle herds over long periods, mainly in clinically healthy animals^22^. The relatively efficient transmission of STEC O157:H7 from calf-to-calf plays an important role in increasing the prevalence of cattle excreting this agent on farms^23^.

Except *stx*_2_, the EHEC O104:H4 outbreak strain lacks other putative STEC-associated virulence and colonization factors^3^. Cattle have not been reported to carry *E. coli* strains belonging to the EAEC pathovar, which is suspected to be adapted to humans^24^,^25^,^26^. Currently available data also suggest that cattle are not the reservoir of the *E. coli* O104:H4 outbreak strain^24^,^27^,^28^. It remains to be determined whether this results from an inability of the strain to colonize cattle, from insufficient exposure of the cattle population to (sufficient numbers of) the bacteria, from insufficiently low frequencies of transmission events, or combinations thereof. It is tempting to speculate that the large 2011 outbreak in the human population affecting several thousand individuals and with an undisclosed number of asymptomatic shedders^29^ could have led to a spillover of the strain to wild-life or livestock populations including cattle. Indeed, we detected genes characteristic of the EHEC O104:H4 strain (*stx*_2_, *aggR*, *wzx*_*O104*_, *fliC*_*H4*_) in fecal pools sampled in a German abattoir processing animals from farms located near the outbreak epicenter two years after the outbreak onset, while contemporarily obtained samples from an unrelated geographical area (Spain) were negative for this combination of genes^30^. The epidemiological evidence to suggest that the outbreak strain has already conquered an animal reservoir is weak. Currently a sound basis for a risk-assessment justifying the establishment or abandoning of targeted preventive measures by veterinary and public health authorities can only come from experimental data generated by infection studies. This prompted the current investigation assessing for the first time if EHEC strains as EHEC O104:H4 can utilize ruminants as reservoir. To this end, we determined the clinical appearance, the pattern and magnitude of fecal shedding and the site of colonization in a well described bovine infection model^31^,^32^.

## Results

### Duration and magnitude of fecal shedding of inoculum strains

**Figure 1:**
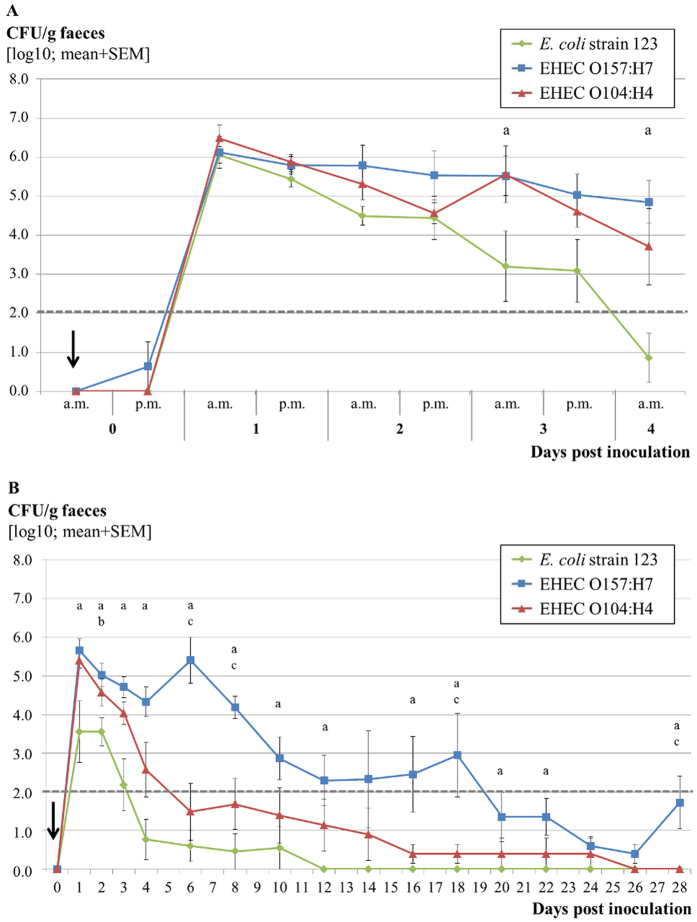
Fecal shedding of *E. coli* by calves inoculated with different strains. Numbers of inoculum bacteria recovered from feces of calves inoculated with EHEC O157:H7, EHEC O104:H4 or *E. coli* strain 123 and followed up for four (Trial 1, N = 5 calves/group; (**A**)) and 28 days post inoculation (Trial 2, N = 5 calves/group; (**B**)) are shown. Data is presented as mean numbers ( ± standard errors of the means) of CFU/g for the indicated groups. Significant differences (Mann-Whitney U-test, p < 0.05) in mean fecal shedding between a: *E. coli* strain 123 and EHEC O157:H7, b: *E. coli* strain 123 and EHEC O104:H4, c: EHEC O157:H7 and EHEC O104:H4. All values below the dashed line represent enrichment levels. The arrow indicates the date of challenge. a.m. = ante meridiem, p.m. = post meridiem.

Fecal samples from all calves inoculated with 10^10^ CFU were culture positive for the respective inoculum strain at several sampling points after inoculation but the magnitude and duration of detectable shedding varied between individuals (Fig. 1).

In Trial 1, fecal shedding of EHEC O104:H4 decreased after an initial peak of >10^5^ CFU/g with a time slope similar to that of the EHEC O157:H7. Both strains were shed by the respective animal groups at approximately 10^5^ CFU/g feces by day 3 pi. EHEC O104:H4 were recovered in higher numbers and for a longer time than the *E. coli* strain 123, which were only detectable in 2 calves at 4 dpi. There is a significant difference (p < 0.05) in shedding between *E. coli* strain 123 and EHEC O157:H7 on days 3 and 4 pi. No significant differences were seen between the numbers of EHEC O104:H4 CFU/g shed in the feces and the respective numbers for *E. coli* strain 123 or EHEC O157:H7.

A second trial conducted for a longer period reproduced the shedding pattern during the first 4 days. Later on, numbers of shed EHEC O104:H4 bacteria decreased dramatically beginning at 4 dpi. Whereas the *E. coli* strain 123 was not detected from 12 dpi onwards, EHEC O104:H4 were detected intermittently until 24 dpi in one calf, on days 16 and 18 pi only by enrichment. Another calf was positive for EHEC O104:H4 by enrichment culture until day 18 pi. EHEC O157:H7 were detected in all 5 calves (2 calves by enrichment culture) until day 12 pi and in 4 calves (2 calves by enrichment culture) until 28 dpi. The remaining calf was positive for EHEC O157:H7 bacteria until day 26 pi by enrichment culture. All 5 calves were negative for EHEC O157:H7 at least at one intermediate sampling date. The differences in fecal shedding between *E. coli* strain 123 and EHEC O157:H7 were statistically significant (p < 0.05, Mann-Whitney test) between days 1 and 28, except on days 14, 24 and 26. There was also a significant difference in shedding between *E. coli* strain 123 and EHEC O104:H4 on day 2 and between EHEC O157:H7 and EHEC O104:H4 on day 6, 8, 18 and 28.

### Distribution of the inoculum strains at necropsy

No gross abnormalities of the mucosa of the forestomaches, the small or large intestines were observed in any of the calves during necropsy at 4 dpi. In Trial 1 EHEC O104:H4 were recovered in similar numbers as EHEC O157:H7 from feces and contents of proximal colon and rectum (RAJ contents) collected from calves inoculated with the respective inoculum strains (Table 1). Significantly lower numbers of the *E. coli* strain 123 were recovered from calves inoculated with *E. coli* 123. EHEC O104:H4 also were recovered from the contents of rumen, abomasum, and gall bladder and from most of the intestinal tissue samples. The numbers of EHEC O104:H4 and EHEC O157:H7 isolated and the number of calves positive for these strains were low within the proximal small intestine and increased in sites distal to the ileum. Distal sites most commonly containing EHEC O104:H4 and EHEC O157:H7 included the ICV, cecum, proximal colon, spiral colon, distal colon, and recto-anal junction (RAJ). EHEC O104:H4 were recovered in equal numbers as EHEC O157:H7. Numbers of EHEC O157:H7 and EHEC O104:H4 significantly differed to bacterial numbers of *E. coli* strain 123 at proximal colon and RAJ. Neither EHEC O104:H4, nor EHEC O157:H7 or *E. coli* 123 were isolated from intestinal lymph nodes, gall bladder (tissue-associated), liver or abomasum (tissue-associated).

Only EHEC O157:H7 were detectable in feces, intestinal content, and associated with intestinal tissue at 28 dpi in Trial 2. EHEC O157:H7 was sporadically recovered from the jejunum (1.6 × 10^3^ CFU/g tissue) and content of proximal colon (10^2^ CFU/g sample) of one calf (#24). EHEC O157:H7 bacteria were also isolated from Peyer´s patches of the ileum (IPP; 4.0 × 10^2^ CFU/g tissue) of one calf (#25), but detected in higher numbers at the RAJ (4.0 × 10^2^ to 4.8 × 10^4^ CFU/g tissue; calf #21, #22, and #25), corroborating the findings at 4 dpi. Duodenum, Peyer´s patches of the jejunum, ICV, caecum, proximal colon, spiral colon, distal colon, content of the rectum, and bile were additionally examined, but negative for the inoculum strains.

### Detection of adherent bacteria by microscopy

**Figure 2:**
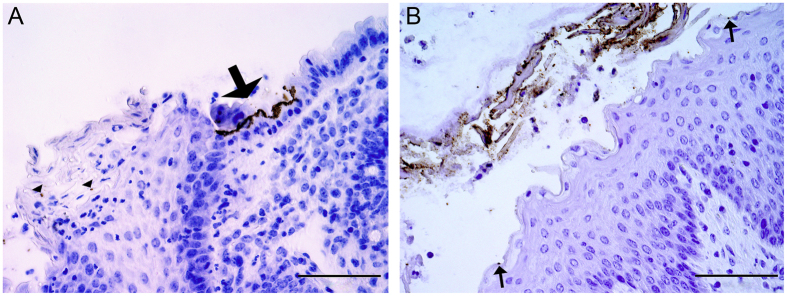
Differences in the localization and pattern of adherence of anti-O157 and anti-O104 positive bacteria at the RAJ from calves necropsied 4 dpi. (**A**) Calf #13, inoculation strain: EHEC O157:H7; O157-positive bacteria form micro-colonies on the epithelium of the rectal mucosa (arrow) and single bacteria are attached to squamous epithelial cells of the RAJ (arrowheads); (**B**) Calf #14, inoculation strain: EHEC O104:H4; numerous O104-positive bacteria surround detached squamous epithelial cells and single O104-positive bacteria are attached to squamous epithelial cells of the RAJ (arrows). Indirect immunoperoxidase for O157 (**A**) and O104 (**B**), bar = 100 μm.

Adherent O157-positive bacteria were observed in defined mucosal sites of all 5 O157-inoculated animals. Single adherent O157-positive bacteria were seen in one calf (#13) at the IPP and in 3 calves (#4, #12 and #13) at the ICV (Supplementary Table 1). In 3 calves (#5, #6, and #13) O157-positive bacteria formed microcolonies on the surface epithelium of the rectal mucosa and single adherent bacteria were present on the squamous epithelium of the RAJ (Fig. 2).

**Figure 3:**
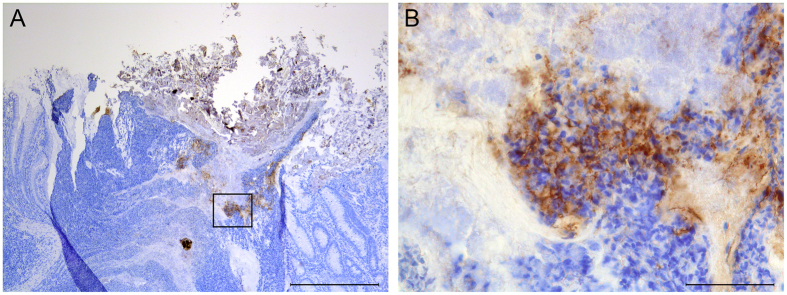
Localization of EHEC O104:H4 at the ICV of calf #14. (**A**) Overview of the dilated crypt of a lymphoglandular complex filled with neutrophils and cellular debris. Multiple groups of O104-positive bacteria are interspersed between neutrophils. Numerous bacteria are also associated with necrotic debris. (**B**) Higher magnification of O104-positive bacteria between neutrophils and attached to necrotic debris. Indirect immunoperoxidase for O104; (**A**) bar = 500 μm, (**B**) bar = 50 μm.

Adherent O104-positive bacteria were seen on the squamous epithelium of the RAJ (Fig. 2) in one (calf #14) of the 5 O104-inoculated calves. The majority of bacteria were associated with detached squamous epithelial cells and single cells were present on the luminal surface of the squamous epithelium. There was no formation of microcolonies and no aggregative adherence. O104-positive bacteria were also found in dilated crypts of lymphoglandular complexes at the ICV that were filled with neutrophils and necrotic debris. Multiple groups of bacteria were seen between the neutrophils and large numbers were attached to the necrotic debris (Fig. 3). In addition, O104-positive bacteria were observed in the neutrophilic exudate of a dilated tonsillar crypt of this calf.

O104- and O157-positive bacterial cells were found in the gut lumen and the intestinal content in every calf inoculated with the respective strain. Large numbers of bacteria were observed on the luminal surface of epithelial cells in some sections of the gastrointestinal tract, but they were not labelled by the primary antibodies used.

### Further characterization of single isolates

**Figure 4:**
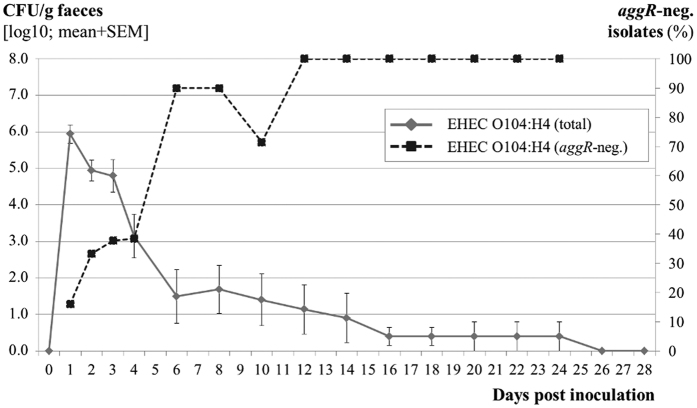
Percentage of aggR-negative isolates of inoculum-type bacteria of strain EHEC O104:H4. (Mean CFU/g feces ± standard deviation, data from all EHEC O104:H4 inoculated calves combined from Trials 1 and 2).

**Figure 5:**
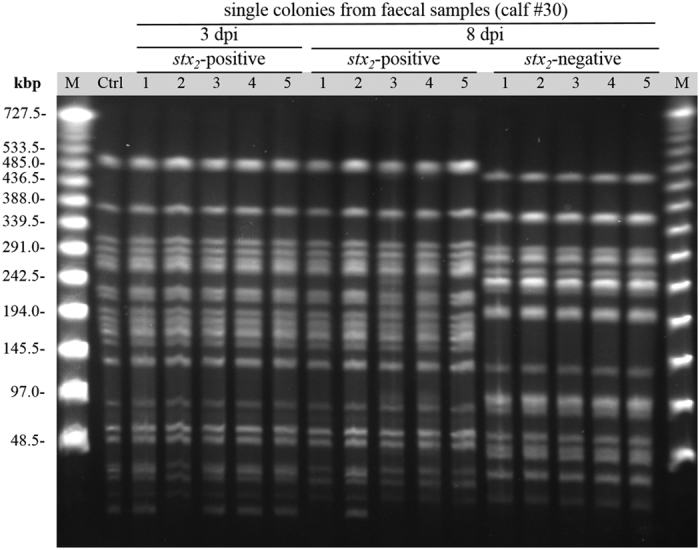
Genetic relatedness of the inoculation strain EHEC O104:H4 and coliform bacteria from fecal samples of calf #30 (Trial 2) taken on days 3 and 8. Overall, *Xba*I-digested DNA of 15 ESBL-positive colonies were analyzed by PFGE that were either putative EHEC O104:H4 re-isolates (*stx*_*2*_- and *aggR*-positive [n = 5; 3 dpi colonies no. 1, 3, 4 and 5 and 8 dpi colony no. 2] and *stx*_*2*_-positive but *aggR*-negative [n = 5] as determined by multiplex PCR [Supplementary Fig. 1]) or other coliform bacteria (*stx*_*2*_-negative, n = 5). 1% TBE agarose gel stained with ethidium bromide. Ctrl: EHEC O104:H4 inoculation strain, M: Lambda Ladder PFG Marker, kbp: kilobases.

Selected colonies recovered from fecal samples, intestinal content, and tissues were verified to be inoculum-type bacteria by multiplex PCR. In total, 315 randomly selected colonies from fecal samples and 189 colonies from intestinal tissues or contents were verified as EHEC O104:H4 inoculum strain by HUSEC041/EAEC multiplex PCR (Supplementary Fig. 1). Ten isolates possessed an additional *astA* gene, which was absent from the strain pre-inoculation. Seven of these colonies were obtained from one calf (#14). All examined isolates of EHEC O104:H4 contained the ESBL gene loci and the pESBL (data not shown). In contrast, 116 of 315 examined EHEC O104:H4 isolates had lost *aggR* (Supplementary Fig. 1) and the corresponding pAA plasmid (data not shown) during passage. The percentage of *aggR*-negative colonies steadily increased during the course of the experiment (Fig. 4). Colony morphology did not differ between *aggR*-positive and -negative colonies. By PFGE analysis of *Xba*I-restricted DNA, two differing DNA fragments of <48.5 kbp length were visible (Fig. 5). The presence of the DNA fragments corresponded to the presence of *aggR* as tested by multiplex PCR in the respective re-isolates (Supplementary Fig. 1).

## Discussion

In 2011, a novel hybrid EHEC strain of serotype O104:H4, possessing a combination of virulence factors of both STEC and EAEC, caused an unprecedented, food-borne outbreak in humans. *E. coli* strains belonging to the EAEC pathovar have not been detected in cattle, the primary STEC/EHEC reservoir thus far. The same holds for hybrid strains like the EHEC O104:H4 outbreak strain even though cattle populations in the outbreak area have intensively been monitored during the outbreak^24^,^27^,^28^. However, as exposure of the cattle population to hybrid EHEC strains like EHEC O104:H4 might occur, the current study aimed at assessing the possibility that strains with this genetic make-up can cross the interspecies barrier from humans to livestock.

The study clearly demonstrated that weaned calves can be colonized by EHEC O104:H4. All calves in these trials shed EHEC O104:H4 following oral administration in numbers indicative of proliferation. Calves had higher fecal levels of inoculated bacteria than had calves inoculated with a non-pathogenic *E. coli* control strain, which is taken as one line of evidence for colonization^16^,^19^. After an initial period of shedding at levels equivalent to the high level shedding of EHEC O157:H7, numbers of shed EHEC O104:H4 declined below the detection limit within 28 days. Calves showed large variation in duration of EHEC O104:H4 shedding, but all calves were culture positive for a minimum of three days. EHEC O104:H4 were detectable for longer periods of time than non-pathogenic *E. coli* 123. One of the experimentally-infected calves even shed EHEC O104:H4 until 24 dpi. These results indicate that a niche in the bovine intestinal tract exists that promotes the replication of EHEC O104:H4 significantly better than that of non-pathogenic *E. coli*, i.e. a stable association between the bacteria and the mucosa.

Accordingly, inoculum-type bacteria were recovered from multiple intestinal sites where *E. coli* strains considered capable of intestinal colonization are found^32^. EHEC O104:H4 bacteria were most frequently detected in the large intestine (e.g. ICV, cecum, proximal colon, spiral colon, distal colon, RAJ). Intestinal levels of inoculum-type bacteria and number of animals positive were similar in EHEC O104:H4 inoculated and EHEC O157:H7 inoculated calves and much higher than in calves inoculated with non-pathogenic *E. coli*. In contrast to EHEC O157:H7, EHEC O104:H4 were not detected at any intestinal site 28 days post inoculation. However, colonization is also defined by the presence of adherent bacteria at 4 dpi^31^,^32^,^33^. We detected adherent bacteria in the RAJ of one calf necropsied 4 days after inoculation with EHEC O104:H4. Bacteria were attached to the surface of squamous epithelial cells. It is conceivable that numbers of EHEC O104:H4 in the other calves of this group were too low or focally distributed to be detected. Adherent bacteria were detected in all calves inoculated with EHEC O157:H7. We confirmed the mucosa-associated lymphoid tissue at the ICV and the rectal mucosa as sites in calves most likely to harbor EHEC O157:H7 A/E-inducing bacteria at 4 dpi^19^,^20^,^21^,^32^. EHEC O157:H7 bacteria were forming microcolonies on enterocytes of the rectal mucosa. The rectum and the RAJ appear to be the principal site of EHEC O104:H4 and EHEC O157:H7 colonization, although there are differences in the mode of attachment and cell types targeted. EHEC O104:H4 were bacteriologically detectable in the contents of the rumen and abomasum. This could be explained by re-infection through general exposures such as contaminated feeds or water sources or by calf-to-calf transmission. It may also be that EHEC O104:H4 is able to adhere to the squamous epithelium of the rumen. We identified the gall bladder as another site where EHEC O104:H4 settled in two calves. Thus, the gall bladder must be considered a possible niche for EHEC O104:H4, similar as for EHEC O157:H7^32^, and may be an additional source of intestinal EHEC O104:H4. At necropsy, the O104-bacteria were also isolated and histologically detected in the tonsils of one calf. This finding may be due to the application of the inoculum (e.g., while retracting the tube after the application) or regurgitation of stomach content and it is not known if occurrence of O104:H4 at this site was only transient. However, EHEC O104:H4 showed an affinity to neutrophils and necrotic debris and because of the structure of the bovine tonsil there may be no need for bacterial attachment at epithelial cells for colonization^34^, introducing tonsils as another biological niche for EHEC O104:H4. Because there was no recovery from lymph nodes (*Lnn. jejunalis*, *Lnn. cecalis*) and liver, EHEC O104:H4 were not invasive in our model.

EHEC O104:H4 bacteria did not cause A/E lesions, as was expected since it lacks the *eae* gene^3^. Surprisingly, this enteroaggregative *E. coli* strain did not show aggregative adherence to bovine intestinal epithelial cells. Bacteria adhered usually as sparse single or few bacilli. Therefore, it is difficult to differentiate them from intestinal material that is labelled non-specifically. The lack of aggregative adherence gives evidence that colonization of the bovine intestine by EHEC O104:H4 does not rely on pAA-encoded adhesion factors. Similar findings were gained in an infant rabbit-based model, where pAA is dispensable for intestinal colonization and development of intestinal lesions^35^. Adherence of EHEC O104:H4 to bovine epithelial cells is perhaps mediated by putative adhesins as the iron‐regulated gene A homologue adhesin (Iha), which is responsible for adherence to epithelial cells in other *eae*-negative STEC^36^. Adherent EHEC O104:H4 were primarily found on squamous epithelial cells of the RAJ. EHEC O157:H7 bacteria have been reported to colonize both glandular and squamous epithelial cells^20^,^32^,^37^, but *in vitro* examination revealed that the respective underlying mechanisms differ^37^. Whether LEE-independent mechanisms to colonize the squamous epithelial cells also differ between EHEC O104:H4 and EHEC O157:H7 remains a hypothetical consideration at present. It also needs to be elucidated, whether EHEC O104:H4 has a general preference for squamous epithelium or if it is limited to lower sections of the bovine gastrointestinal tract. In this study, EHEC O104:H4 were also found in dilated crypts of lymphoglandular complexes and dilated tonsillar crypts that were filled with neutrophils and necrotic debris. EHEC O104:H4 may use aggrieved parts of the mucosa as another biological niche.

Analyses of sequential fecal samples demonstrated that EHEC O104:H4 are subject to a steady change of variable genetic elements. The virulence plasmid of enteroaggregative *E. coli* (pAA) is a relatively unstable genetic element as demonstrated by its intra-host loss during course of human infection^38^. A role of the pAA for adherence of EHEC O104:H4 to the bovine intestine remains obscure, because *agg*R-positive EAEC have rarely been detected in cattle^30^,^39^. pAA-negative derivatives isolated from humans lost their ability to efficiently colonize the intestinal epithelium, which results in lack of systemic absorption of Stx2 and diminished the ability of EHEC O104:H4 to cause HUS^38^. In this study, the adherence patterns of EHEC O104:H4 suggest that pAA is not necessary for colonization of bovine epithelium. Moreover, the selection of pAA-negative EHEC O104:H4 in bovines may lead to an enrichment of strains with reduced colonization efficiencies in humans. In contrast to the lability of pAA, the *stx*_2a_ gene of EHEC O104:H4, which is encoded on an inducible bacteriophage^40^, and the ESBL plasmid were stable during bovine infection as demonstrated by their presence in all isolates and as reported for human isolates^38^. Some isolates even acquired the *astA* gene encoding EAST1 (enteroaggregative *Escherichia coli* heat-stable enterotoxin 1), which is a small protein first detected in EAEC, but is present also in other pathovars of intestinal *E. coli*^41^. Strains expressing EAST1 have been shown to induce diarrhea principally in humans, but have also been found in piglets and calves^42^. Beside the loss of the pAA plasmid no other genetic modifications were detectable in the re-isolates. By PFGE analysis only small DNA fragments differed between *aggR*-positive and -negative re-isolates corresponding in their molecular size to *Xba*I-fragments of the pAA plasmid as calculated by the plasmid DNA sequence (PubMed accession no. CP011332).

Inoculation of calves with 10^10^ CFU of EHEC O104:H4 did not induce significant clinical disease. The transient non-bloody diarrhea noted in some calves may have been due to fasting before inoculation or endotoxin absorption associated with the administration of massive numbers of gram-negative bacteria^33^. Spontaneous, transient diarrhea occurs occasionally in otherwise healthy experimental calves^43^. Although being fairly non-pathogenic for cattle similar to classical STEC strains, EHEC O104:H4 appear to be less well adapted to bovine intestine than classical STEC strains. EHEC O104:H4 was not yet found in cattle herds^27^,^28^,^44^ but the present experimental infection study, as well as epidemiological evidence gathered in the outbreak region^30^, support the hypothesis that EHEC O104:H4 and probably other EAEC may be or become part of the *E. coli* microbiome in this livestock species and contribute to and participate in the genome plasticity through uptake and loss of virulence genes^45^. Future risk assessments must therefore take cattle as an animal reservoir for EHEC/EAEC hybrid strains and a potential source of transmission to humans into account.

## Methods

### Bacterial strains and inocula

EHEC O104:H4 strain LB226692 (Table 2) was isolated during the outbreak 2011 in Germany^3^ and fully characterized by whole genome sequencing^4^. The strain has an ESBL phenotype^3^. EHEC O157:H7 strain 86–24 is a nalidixic acid-resistant mutant of an Stx2-producing strain isolated from an outbreak in Washington state^46^ and was used as a positive control. A nalidixic acid resistant mutant of *E. coli* strain 123 (O43:H28), a porcine isolate which does not produce Stx and is not pathogenic in cattle^47^, was used as a negative control strain. For ease of reading, strain 123 is referred to as “*E. coli* strain 123”, strain 86–24 Nal as “EHEC O157:H7” and strain LB226692 as “EHEC O104:H4” throughout the manuscript. Stock inocula containing approximately 10^10^ CFU/ml were prepared as previously described^31^ and stored in 1 ml aliquots at −80 °C until used. Actual numbers of viable EHEC O104:H4 bacteria in each thawed batch were confirmed by bacterial colony counts on Brilliance ESBL agar plates (Oxoid, Basingstoke, United Kingdom), whereas EHEC O157:H7 and the *E. coli* strain 123 were quantitated on MacConkey agar containing 50 mg of nalidixic acid per ml.

### Animals and animal treatments

A detailed description of the animal model is given in the Supplementary Methods. In brief, weaned female Holstein calves were housed in an environmentally controlled animal facility at the Friedrich-Loeffler-Institut (FLI) on the Isle of Riems, Greifswald. The experimental protocol was reviewed by an independent animal welfare and ethics committee, approved by the local authority (State Office for Agriculture, Food Safety and Fisheries of Mecklenburg-Western Pomerania, Rostock, Germany, reference no. 7221.3-1.1-093/12), and carried out in accordance with the approved guidelines.

All calves were clinically normal at the time of inoculation. Calves (five/group) were inoculated intra-rumenally with10^10^ CFU of the particular strain.

In the short-term experiment (Trial 1), calves were observed twice a day when fecal samples were taken (0 to 4 days post inoculation [dpi]). Peripheral venous blood samples were collected pre-inoculation and every second day. Necropsy and sampling were performed at 4 dpi as previously described^48^. In the long-term experiment (Trial 2) calves were observed twice a day and fecal samples were collected daily in the morning for the first four days (0 to 4 dpi) and then every second day (6 to 28 dpi). Peripheral venous blood samples were collected pre-inoculation and once a week. Necropsy and sampling were performed at 28 dpi.

### Bacteriologic examination

A comprehensive description is given in the Supplementary Methods. Briefly, fecal samples were obtained from all calves and screened for inoculum-type bacteria and antibiotic-resistant normal flora by plating on selective media used for quantitating the inoculum strains. Selected colonies were tested for O43, O104, and O157 antigens by slide agglutination using appropriate antigen-specific sera (*E. coli* O43 antiserum, *E. coli* K9 serum^49^, *E. coli* OK O157 antiserum; Serum Statens Institut, Denmark). For isolation of single *stx*_2_-positive colonies, colony hybridization was done as previously described^22^. Up to 5 *stx*_2_-positive and 5 *stx*_2_-negative colonies per sample were sub-cultured and stored with 30% glycerine.

### Histologic studies

Tissues were fixed in NBF for 24 to 48 h, embedded in paraffin, sectioned, and stained with hematoxylin and eosin for routine histology. Bacteria were identified in formalin-fixed intestinal tissues by indirect immunoperoxidase method using rabbit *E. coli* OK O157 antiserum and rabbit *E. coli* K9 serum^49^ (Serum Statens Institut, Denmark) as the primary antibodies and peroxidase-conjugated anti-rabbit IgG antiserum (Jackson ImmunoResearch Laboratories Inc., West Grove, USA) as secondary antibody. Antigens were retrieved by trypsin digestion^50^. Sections were counterstained with Mayer´s hemalum.

### Further characterization of single isolates

Multiplex PCR targeting typical molecular features of EHEC O104:H4 (*rfb*_*O104*_^3^, *fliC*_*H4*_^3^, *stx*_2_^51^, *terD*^52^, *aggR*^53^, *pic*^54^, and *astA*^55^) and EHEC O157:H7 (*rfb*_*O157*_^56^, *fliC*_*H7*_^57^, *stx*_2_^58^, and *terD*^52^) was used for further characterization of the stored cultures. Presence of the ESBL gene loci (*blaTEM*^59^, *blaCTX-M*^60^, and *blaCTX-M-15*^61^) was also examined by multiplex PCR. Multiplex PCR was done with the PANScript DNA-Polymerase (PAN-Biotech GmbH, Aidenbach, Germany) in a total volume of 27 μL containing 3 μL of bacterial DNA. PCR conditions included initial denaturation at 95 °C for 1 min, 35 cycles of denaturation (95 °C, 30 s), annealing (55 °C, 60 s), and extension (72 °C, 60 s), and final extension at 72 °C for 5 min. Contour-clamped homogeneous electric field-pulsed-field gel electrophoresis (CHEF PFGE) of *Xba*I-digested DNA was performed as previously described^62^.

### Statistical analysis

Statistical analysis was done with “SPSS for Windows” (Version 23, SPSS Inc., Chicago, Illinois, U.S.A.). The Mann-Whitney test was used to determine the significance of differences between the numbers of bacteria recovered from the three different inoculation groups. Two-tailed p- values with *p* ≤ 0.05 were considered significant.

## Additional Information

**How to cite this article**: Hamm, K. *et al*. Experimental Infection of Calves with *Escherichia coli* O104:H4 outbreak strain. *Sci. Rep*. **6**, 32812; doi: 10.1038/srep32812 (2016).

## Supplementary Material

Supplementary Information

## Figures and Tables

**Table 1:** Recovery of the inoculum strains from calves at necropsy 4 dpi (Trial 1).

Strain	CFU/g sample [log10]^a^																		
	Calf no.	Gastrointestinal tissues											Intestinal content						
		Tons	Duo	Jej	JPP	IPP	ICV	Cec	PC	SC	DC	RAJ	Fec	PC	RAJ	Rum	Abo	GB	
*E. coli*	1	0	0	0	0	0	0	0	0	0	0	0	0	2.5	0	2.0	0	0	
strain 123	2	0	0	0	0	0	0	0	0	0	0	0	0	0	4.3	0	0	0	
	3	0	0	0	0	0	0	0	0	0	0	0	0	0	4.2	0	0	0	
	10	0	0	0	4.1	2.3	4.5	3.4	3.0	4.0	0	0	1	0	0	0	0	0	
	11	0	0	0	0	0	3.6	3.9	0	0	0	0	3.2	2.3	3.3	0	0	0	
EHEC	4	0	0	0	0	2.8	3.9	2.9	4.8	2.3	2.3	6.1	6.0	3.2	5.9	2.8	0	0	
O157:H7	5	0	0	n.t.	0	2.8	6.4	3.0	4.1	3.5	3.7	6.0	5.6	4.4	6.2	2.9	0	0	
	6	0	0	0	0	0	0	0	0	0	0	5.8	5.1	4.2	4.4	0	0	0	
	12	0	2.6	0	2.8	0	7.6	5.4	4.6	4.9	4.3	5.3	2.9	3.7	3.0	0	0	0	
	13	4.2	0	0	0	7.0	7.0	7.1	6.7	7.1	5.6	7.5	4.6	5.1	5.5	0	0	0	
EHEC	7	0	0	0	0	0	7.1	6.8	7.0	4.9	4.6	6.8	5.8	5.6	7.1	2.0	0	3.2	
O104:H4	8	0	0	3.7	0	0	0	0	0	0	0	0	0	5.2	7.8	2.7	0	3.2	
	9	0	0	0	0	0	5.0	4.8	5.2	2.7	4.1	0	4.5	5.3	5.7	2.8	6.0	0	
	14	2.5	5.1	0	2.9	3.0	4.8	5.9	3.9	4.6	0	4.5	4.1	3.7	3.5	0	0	0	
	15	0	0	4.5	5.8	5.6	4.4	6.4	5.4	5.4	5.4	4.8	4.2	4.4	5.1	0	0	0	
significantly different mean bacterial load^b^		—	—	—	—	—	—	—	I, II	—	I, II	I	I	I, II	II	—	—	—	

^a^Abbreviations: Abo = Abomasum, Cec = Caecum, CFU = colony forming units, DC = distal colon, dpi = days post inoculation, Duo = Duodenum, Fec = Feces, GB = gall bladder, ICV = ileocecal valve, IPP = Peyer’s patches of the ileum, Jej = Jejunum, JPP = Peyer’s patches of the jejunum, n.t. = not tested, PC = proximal colon, RAJ = rectoanal junction, Rum = Rumen, SC = spiral colon, Tons = Tonsils.

^b^Significant differences of the mean bacterial loads [CFU/g sample] between (I) group *E. coli* 123 and group EHEC O157:H7, (II) group *E. coli* 123 and group EHEC O104:H4 or (—) no significant difference between the groups (Mann-Whitney test, p ≤ 0.05).

**Table 2:** *Escherichia coli* strains used in this study.

Strain	Serotype	Pathotype^a^	Virulence factors/ genotypic features^b^	antibiotic resistence^c^	Source^d^	Reference
123	O43:H28	none	none	Nal^R^	NADC	47
86–24 Nal	O157:H7	EHEC	*stx2a*, LEE	Nal^R^	NADC	46
LB226692	O104:H4	EHEC	*stx2a*, EAEC virulence plasmid (pAA; 83 kbp; *aatA*, *aggR*, *aaf/I*), additional plasmid (1.5 kbp)	Nal^R^, Strep^R^, ESBL plasmid (pESBL; 90 kbp; *blaTEM*-*1*, *blaCTX*-*M-15*)	IHM	4

^a^EAEC = enteroaggregative *E. coli*, EHEC = enterohemorrhagic *E. coli*.

^b^ESBL = Extended-Spectrum Beta-Laktamase, LEE = locus of enterocyte effacement.

^c^Nal = Nalidixic acid, ^R^ resistance, Strep = Streptomycin.

^d^IHM = Institute of Hygiene, University of Münster, NADC = National Animal Disease Center, Agricultural Research Service, U.S. Department of Agriculture.
