# Supplementary material for: Experimental Infection of Calves with Escherichia coli O104:H4 outbreak strain
Source: Sci Rep. 2016 Sep 7;6:32812. doi: 10.1038/srep32812 (PMC5013450; doi:10.1038/srep32812)
Supplement: Supplementary Information [file srep32812-s1.doc]

# Experimental Infection of Calves with *Escherichia coli* O104:H4 outbreak strain

K. Hamm, S.A. Barth, S. Stalb, L. Geue, E. Liebler-Tenorio, J.P. Teifke, E. Lange, K. Tauscher, G. Kotterba, M. Bielaszewska, H. Karch, and C. Menge

# Supplementary Table 1 │ Immunohistochemical detection of adherent O157- or O104-positive bacteria

# Samples obtained from weaned calves 4 days after inoculation with EHEC O157:H7 and EHEC O104:H4

|  | No. of animals positive/tested for: | |
| --- | --- | --- |
| Localization | EHEC O157:H7 | EHEC O104:H4 |
| Duodenum | 0/5 | 0/5 |
| Jejunum | 0/5 | 0/5 |
| Peyer´s patches in the jejunum | 0/5 | 0/5 |
| Peyer´s patches in the ileum | **1**/5 | 0/5 |
| Ileocecal valve | **3**/5 | 0/5 |
| Cecum | 0/5 | 0/5 |
| Proximal colon | 0/5 | 0/5 |
| Ansa centralis | 0/5 | 0/5 |
| Distal colon | 0/5 | 0/5 |
| Rectoanal junction | **3**/5 | **1**/5 |
| Gall bladder | 0/5 | 0/5 |

# Supplementary Figure 1 │ Agarose gel image of amplicons obtained from a HUSEC041/EAEC multiplex PCR of *stx_2_*-positive colonies from fecal samples of calf #30 (Trial 2) on day 3 and 8.

# 2 % TBE agarose gel stained with ethidium bromide. Controls: +1: EHEC O104:H4, +2: HUSEC041, ‑: medium, M: DNA marker GeneRuler™ 100 bp Plus DNA Ladder, bp = base pairs.


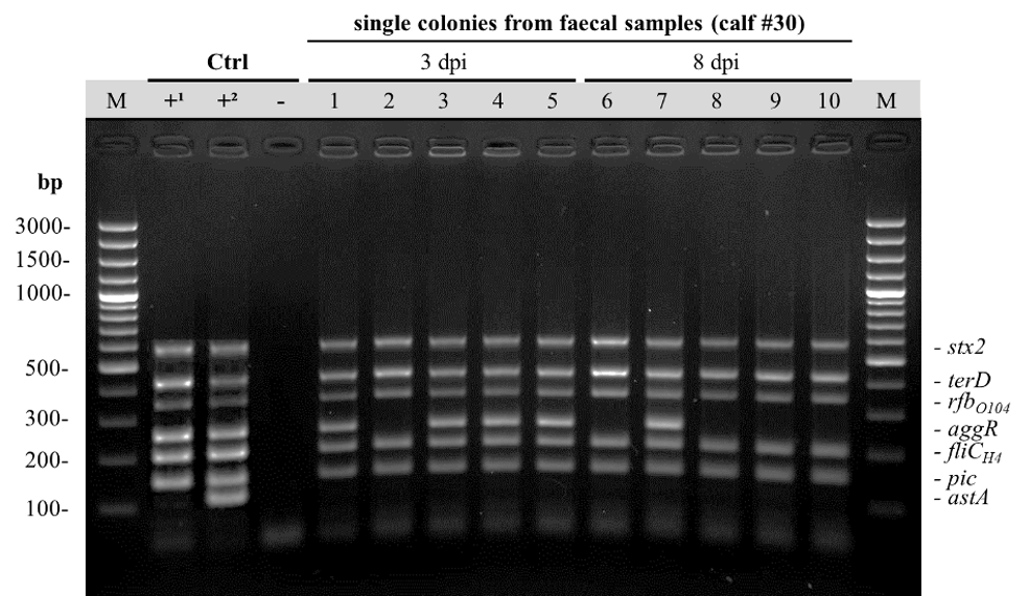


# Supplementary Methods

## Animal Model

## Animals. Weaned female Holstein calves, aged 87 - 137 days (body weight mean: 118.6 kg) were housed in an environ­mentally controlled animal facility at the Friedrich-Loeffler-Institut (FLI) on the Isle of Riems, Greifswald for at least 1 week prior to inoculation and for the duration of the study. Calf inoculation experiments were performed in isolation rooms, where the calves of one inoculation group were placed in one pen together. The calves were fed a diet of grain and hay appropriate for their age throughout the experiment. All calves were tested for intestinal pathogens (*Salmonella* spp., *Yersinia* spp., *Eimeria*spp., *Trichuris* spp.) and for pre-inoculation STEC (*stx_2_*, *stx_1_*[^1^](#_ENREF_1)) and EAEC (*aggR*[^2^](#_ENREF_2)) shedding by multiplex PCR.

Inoculum-type bacteria were not recovered by direct culture from any of the pre-inoculation fecal samples. Four calves were *stx*_1_ positive, 9 calves *stx*_2_ positive and 6 calves *stx*_1_*/stx*_2_ positive in feces before inoculation. The *aggR* gene was not detected in feces of any of the calves. None of the calves shed detectable levels of *Salmonella* spp*.* or *Yersinia* spp*.*, but 23 calves were positive for *Eimeria* spp. and 11 calves for *Trichuris* spp*.*.

## Animal treatments and inoculations. All calves were clinically normal at the time of inoculation. To enhance their susceptibility to enteric *E. coli* infection, all calves were fasted for 48 h before they were inoculated. Calves (five/group) were inoculated intra-rumenally (with a stomach tube) with approximately 10^10^CFU of the particular strain resuspended in 200 ml of Trypticase soy broth (TSB) (Sifin Diagnostics GmbH, Berlin, Germany), followed by 200 ml of TSB.

In the short-term experiment (Trial 1), calves were observed twice a day when fecal samples were taken (0 to 4 dpi) and stored at 4 °C for bacteriological culture. Peripheral venous blood samples were collected pre-inoculation and every second day. Necropsy and sampling were performed at 4 days post inoculation (dpi) as previously described[^3^](#_ENREF_3). Briefly, calves were pre-medicated with 0.1 mg xylazine hydrochloride/kg of body weight (BW) and anesthetized with 4 to 8 mg ketamine/kg of BW intravenously. The abdominal cavity was opened and the intestinal convolute exposed. Loops of approximately 10-cm lengths were tied in the duodenum, mid jejunum, jejunum containing a Peyer’s patch (JPP), ileum with Peyer´s patch (IPP), and spiral colon, and each loop was filled with 4 % neutral buffered formalin (NBF). The calves were then euthanized with 50 mg pentobarbital sodium/kg BW intravenously. Intestinal tissue and segments from the ileocecal valve (ICV), cecum, proximal colon, distal colon, and rectoanal junction (RAJ) were collected, opened at the mesenteric attachment, pinned flat on polystyrene, and immersed in NBF for 24 h. The remaining intestines were detached from the mesentery and opened, and the intestinal content and intestinal wall were inspected. Tissues from duodenum, mid jejunum, JPP, IPP, ICV, cecum, proximal colon, spiral colon, distal colon and RAJ, abomasum, gall bladder, jejunal lymph nodes, cecal lymph nodes, tonsils, and liver as well as contents from the proximal colon, RAJ, rumen, abomasum, and gall bladder were collected aseptically and stored at 4 °C for bacteriological culture. A complete necropsy was performed, and samples collected from palatine tonsil, spleen, jejunal lymph node, cecal lymph node, popliteal lymph node, lung, heart, liver, kidney, gall bladder, rumen, and abomasum were fixed in NBF.

In the long-term experiment (Trial 2) calves were observed twice a day and fecal samples were collected daily in the morning for the first four days (0 to 4 dpi) and then every second day (6 to 28 dpi). Peripheral venous blood samples were collected pre-inoculation and once a week. All calves were stunned with a captive bolt pistol, exsanguinated, and necropsied at 28 dpi. Only bacteriological examination of aseptically-collected sections of tissues and intestinal content was done.

All calves remained healthy throughout the study with the exception that 13 calves (4 inoculated with the *E. coli* strain 123, 5 with EHEC O157:H7, 4 with EHEC O104:H4) had transient (< 24 h) episodes of non-bloody diarrhea within 24 h of inoculation and 11 calves (5 inoculated with *E. coli* strain 123, 2 with EHEC O157:H7, 4 with EHEC O104:H4) developed diarrhea sporadically during the experiment, which lasted for one or two days.

## Bacteriologic examination. At 35 days before (i.e. at the farm of origin) to 1 day after housing in the animal facility at FLI, fecal samples were obtained from all calves and screened for inoculum-type bacteria and antibiotic-resistant normal flora by plating on selective media used for quantitating the inoculum strains. Freshly isolated fecal samples (approximately 1 g) were diluted 1:10 and serially diluted in sterile phosphate-buffered saline. Selective agar plates were used as described above. Enrichment cultures were performed by adding up to 0.5 g of feces to 5 ml trypticase soy broth. These cultures were incubated overnight at 37 °C without shaking. The detection limit of direct plating was 100 CFU/g. Samples from enrichment cultures of feces that were negative by direct plating were incubated on Brilliance ESBL or MacConkey agar containing 50 mg of nalidixic acid per ml. Enrichment plates were scored as either positive or negative without counting colonies. Positive enrichment cultures were arbitrarily assigned a value of 10 CFU/g, and those which were negative were given an arbitrary value of 1 CFU/g for graphic presentation.

Selected colonies were tested for O43, O104, and O157 antigens by slide agglutination using appropriate antigen-specific sera (*E. coli* O43 antiserum, *E. coli* K9 serum[^4^](#_ENREF_4), *E. coli* OK O157 antiserum; Serum Statens Institut, Denmark). For isolation of single *stx*_2_-positive colonies, colony hybridization was done as previously described[^5^](#_ENREF_5). Briefly, colonies were blotted on a nylon membrane disk (Roche Diagnostics GmbH, Mannheim, Germany). After lysis of the colonies, removal of cell debris and fixation of DNA, DIG Easy Hyb solution (Roche Diagnostics GmbH, Mannheim, Germany) was used for pre-hybridization of nylon membranes. For hybridization, DNA-probes labeled with digoxigenin (DIG) using the PCR DIG Probe Synthesis Kit (Roche) with *stx*_2_-primers[^1^](#_ENREF_1) were added to DIG Easy Hyb. DIG-labeled *stx*_2_-positive colonies were detected by enzyme immunoassay using sheep anti-DIG-AP Fab fragments and enzyme-catalyzed color reaction with 4-nitro blue tetrazolium chloride (NBT, Roche) and 5-bromo-4-chloro-3-indolyl-phosphate (BCIP, Roche). Up to 5 positive and 5 negative colonies per sample were selected, sub-cultured in Nutrient Broth I (Sifin Diagnostics, Berlin, Germany), incubated overnight at 37 °C and stored with 30 % glycerine at ‑80 °C for further characterization.

# Supplementary References

1. Müller D*, et al.* Identification of unconventional intestinal pathogenic *Escherichia coli* isolates expressing intermediate virulence factor profiles by using a novel single-step multiplex PCR. *Appl Environ Microbiol* **73**, 3380-3390 (2007).

2. Boisen N, Struve C, Scheutz F, Krogfelt KA, Nataro JP. New adhesin of enteroaggregative Escherichia coli related to the Afa/Dr/AAF family. *Infect Immun* **76**, 3281-3292 (2008).

3. Otto PH, Clarke IN, Lambden PR, Salim O, Reetz J, Liebler-Tenorio EM. Infection of calves with bovine norovirus GIII.1 strain Jena virus: an experimental model to study the pathogenesis of norovirus infection. *J Virol* **85**, 12013-12021 (2011).

4. Kogan G, Jann B, Jann K. Structure of the *Escherichia coli* O104 polysaccharide and its identity with the capsular K9 polysaccharide. *FEMS Microbiol Lett* **91**, 135-140 (1992).

5. Geue L*, et al.* A long-term study on the prevalence of shiga toxin-producing Escherichia coli (STEC) on four German cattle farms. *Epidemiology and infection* **129**, 173-185 (2002).
